# A Geranylated Natural Product Simamycin Disrupts the Allosteric Catalysis of tRNA-2-selenouridine Synthase SelU

**DOI:** 10.1021/acs.biochem.5c00053

**Published:** 2025-05-26

**Authors:** Stephen J. Dansereau, Alexander Shekhtman, Francesco Epifano, Salvatore Genovese, Serena Fiorito, Thomas J. Begley, Jia Sheng

**Affiliations:** † Department of Chemistry, University at Albany, 1084 State University of New York, 1400 Washington Avenue, Albany, New York 12222, United States; ‡ The RNA Institute, University at Albany, State University of New York, 1400 Washington Avenue, Albany, New York 12222, United States; § Department of Pharmacy, University “Gabriele D’Annunzio” of Chieti-Pescara, Chieti Scalo 66100, Italy; ∥ Department of Biological Science, University at Albany, State University of New York, 1400 Washington Avenue, Albany, New York 12222, United States

## Abstract

tRNA-2-selenouridine synthase (SelU) is a tRNA-modifying enzyme that is instrumental to bacterial translation by exploiting certain chalcogens. Specifically, this enzyme catalyzes the geranylation of 2-thiouridine at the wobble position of three bacterial tRNAs to enhance the recognition of codons ending in guanosine over adenosine using geranyl pyrophosphate as the cofactor. In addition, SelU is also the working enzyme for a selenation process at the same tRNA position in the presence of selenophosphate. How this enzyme conducts two mechanistically different reactions is a fundamentally interesting question. In order to gain more details about the substrate recognition of SelU, in this work, we identified a small natural compound simamycin (5′-*O-*geranyluridine) with strong interactions with this enzyme. Further, through biophysical affinity assays and NMR structural studies, we postulated an allosteric mechanism of SelU catalysis involving cooperativity among each domain and a conformational rearrangement around the active site of its N-terminal domain. This conclusion is supported by the bimolecular quenching constants, number of binding sites, and thermodynamic parameters calculated for this compound complexed with the N-terminal domain of SelU.

## Graphical abstract

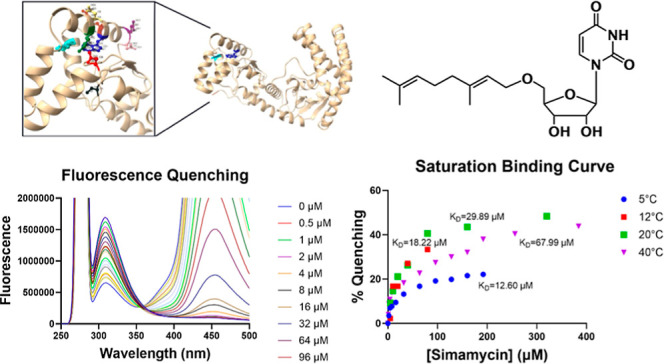

## Introduction

RNA is widely modified to diversify its structures and functions. Over 170 naturally modified nucleotides have been identified in different RNA systems, mainly in mRNA, tRNA, rRNA, and other noncoding RNAs.^1,2^ These RNA modifications play important roles in fundamental biochemistry and therefore have great significance in gene regulation and therapeutic development.^3–5^ The association between tRNA modifications and many diseases including viral infections and cancers has been well established.^5^ These nonclassical residues have been shown to affect both fidelity and efficiency of codon–anticodon recognition, enhance ribosomal binding, fine-tune tRNA structures, and regulate gene stability and expression.^5–7^ The first anticodon position, or wobble position 34 is most frequently modified with a wide range of chemical groups that are directly involved in the tRNA decoding and translation processes.^6,8–10^ One of these common modifications at the wobble position is 2-thiouridine (s^2^U) and its derivatives (Figure 1). The chemical groups on position 5 can enhance ribosomal binding and codon recognition to both adenosine (A)- and guanosine (G)-ending codons.^6,9^

**Figure 1:**
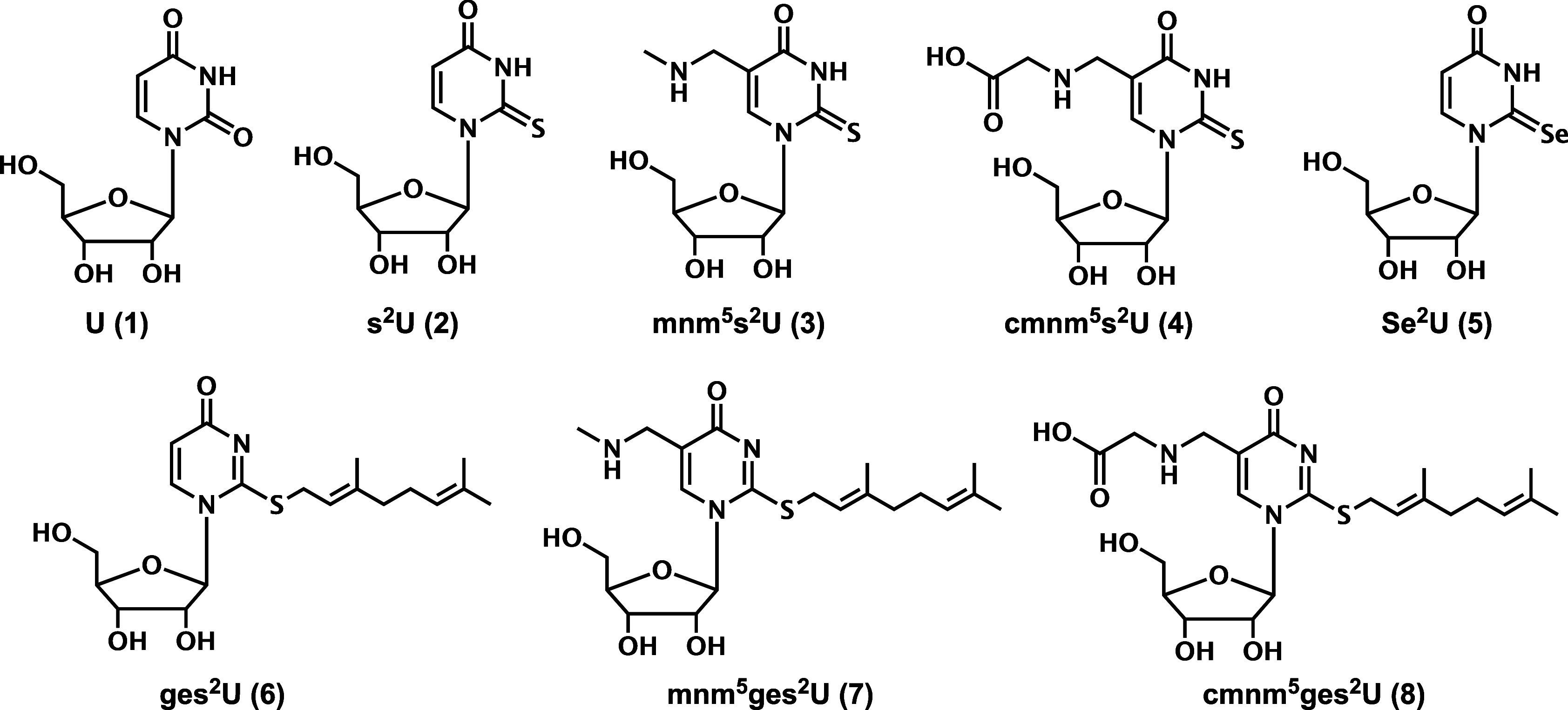
Chemical structures of uridine (1), 2-thiouridine derivatives (2–4), 2-selenouridine (5), and 2-thio-*S*-geranyluridine derivatives (6–8).

The 2-thiolation of uridine has been discovered on 16 out of 60 natural modified uridines^1^ and has been shown to enhance the U–A interaction while decreasing the U–G recognition.^11–13^ In addition, the 2-sulfur atom could be further replaced by selenium (compound 5 in Figure 1), catalyzed by tRNA 2-selenouridine synthase (SelU, also called MnmH).^14^ Interestingly, the same enzyme can also install a geranyl group onto the sulfur atom at the same position 2 using geranyl-pyrophosphate as the cofactor, generating a series of geranyluridine derivatives (compounds 6–8 in Figure 1).^8,15–17^ How SelU conducts the bifunctions of both selenation and geranylation is a fundamentally interesting question. It has been reported that the geranylation level was reduced as the in vitro level of selenium substrate exceeded 10 nM,^8^ implying that geranyl-2-thiouridine might be an intermediate from 2-thiouridine to 2-selenouridine.^18,19^ This, of course, calls into question how the varying in vivo selenide concentrations across bacteria in different geochemical environments direct catalysis.^20,21^ Nonetheless, other than merely being the selenouridine intermediate, the geranyluridine might have other roles, such as facilitating tRNA localization, translational regulation, and the cellular stress response. We previously synthesized the geranyl-modified DNA and RNA oligonucleotides and investigated the importance of the geranyl group in base pairing stability and specificity.^22–24^ The geranyl modification has been found in tRNAs specific for glutamate, glutamine, and lysine in many bacteria including Escherichia coli, Enterobacter aerogenes, Pseudomonas aeruginosa, and Salmonella enterica var. Typhimurium. The geranylated-tRNA^Lys^ has been reported to reduce −1 frameshifting during the translation of E. coli genes, while geranylated-tRNA^Glu^ promotes the codon bias of GAG to GAA.^8^ In addition, the aminoacyl tRNA_Se_^Lys^ and tRNA_Se_^Glu^ show increased recognition of codons ending in G, compared to their aminoacyl tRNA_S_ counterparts, suggesting a role in translation.^25^

All these data indicate the biological significance of SelU in bacterial growth and its potential as an antibiotic target. This 364 amino acid protein is divided into an N-terminal rhodanese domain^26^ containing the tRNA binding site and a C-terminal P-loop domain thought to bind ATP^27^ and geranyl pyrophosphate (GePP).^28^ In fact, purified SelU contains a tightly bound tRNA, and incubation of bovine rhodanese with selenite and glutathione yields a rhodanese-selenium adduct.^29^ The selenation reaction does not occur if either ATP or tRNA is omitted, even when SelU is present.^30^ SelD knockdown experiments by Veres and Stadtman^31^ revealed that this selenium donor is produced by selenophosphate synthetase reacting with ATP and selenide, while no effect of ATP was noted on ^75^Se incorporation into tRNA, indicating the sole role of ATP being the generation of selenophosphate. Similar to this selenophosphate-mediated substitution, geranyl pyrophosphate reacts with 2-thiouridine tRNA to generate S-geranylated tRNA. Szczupak et al. demonstrated that this reaction is chain-length-specific, particularly favored by longer prenyl groups such as geranyl or farnesyl isoprenoids.^32,33^ In order to gain more detailed insights into the substrate recognition of SelU, in this work, we identified a small natural compound called simamycin (5′-*O-*geranyluridine, Figure 2A, left) that has a strong interaction with this enzyme as a substrate mimic of geranyl pyrophosphate (GePP, Figure 2A, right). Through biophysical affinity assays and NMR structural studies, we characterized the receptor–ligand complex in terms of the interacting residues of the enzyme and the complementary functional groups of the ligand. We also postulated an allosteric mechanism of SelU catalysis involving cooperativity among each domain and a conformational rearrangement around the active site of its N-terminal domain, which is supported by the bimolecular quenching constants, number of binding sites, and thermodynamic parameters calculated for this compound complexed with the N-terminal domain of SelU.

**Figure 2:**
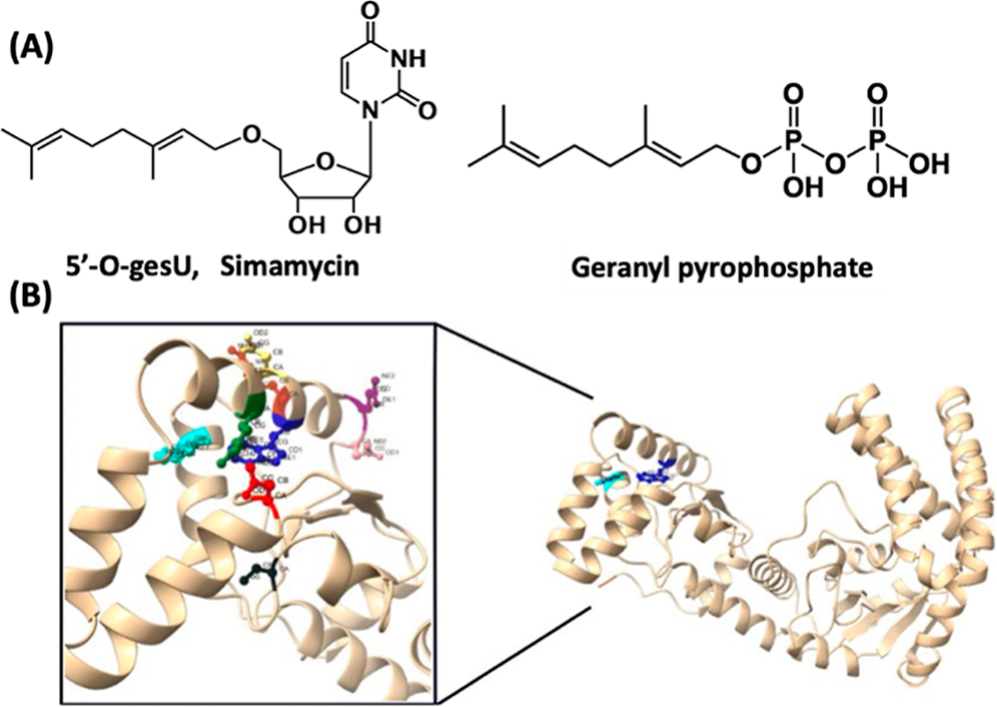
(A) Chemical structures of geranylated natural products simamycin and geranyl pyrophosphate. (B) Alphafold-generated 3D structure of M1-A172 SELU. The polar side chains of residues within 6 Å of W83 include N44 (red), R79 (green), D81 (yellow), R84 (orange), Q89 (purple), N90 (pink), and W110 (turquoise). C97 (black) is just beyond this vicinity.

Simamycin is a unique uridine ribonucleoside with an O-geranyl group branching from the sugar as a 5′-CH_2_O-ether. The former imitates the geranyl pyrophosphate cofactor, while the latter provides dipole moments and hydrogen bond donors and acceptors, typical of nucleosides. This ribonucleoside belongs to a rare group of naturally occurring products called “nucleoterpenes”. These are extremely rare phytochemicals for which only a few examples about their isolation and structural characterization have been reported in the literature. One such product is avinosol, an N^1^-sesquitertpeny-2′-deoxyinosine derivative isolated from Dysidea sponges, and having been shown to exert valuable and promising antiangiogenic and antimetastatic effects.^34^ Other examples are JBIR-68 and farnesides A and B, three dihydrouracil nucleosides carrying geranyl and farnesyl chains linked to the ribose core, isolated from Streptomyces spp. strain RI-18 ^35^ and CNT-372 ^36^ and exhibiting anti-H1N1 virus activity and mild antiplasmodial effects, respectively. Simamycin has been isolated for the first and up to now the only time from Streptomyces spp., a soil-derived strain TP-A0872, and shown to strongly induce preadipocyte differentiation into mature adipocytes.^37^ Given the structural resemblances of simamycin to native SelU ligands and the known biological activities of its broader class of nucleoterpenes, we believed that this natural product could provide a molecular framework to be further optimized into a SelU inhibitor. In doing so, we speculate how this natural geranyl-containing compound might interact with SelU as a mimic of 2-S-geranyl uridine.

## Results and Discussion

A properly chosen pharmacologic scaffold should offer a stereochemistry and interacting moieties complementary to the target. We sought to simplify our ligand-based approach to designing a small-molecule inhibitor; two proposed scaffolds based on their geranyl groups are illustrated in Figure 2A, targeting SelU by investigating the purported tRNA-binding domain as a recombinant protein. Since the three-dimensional structure of SelU is yet to be determined experimentally, this shorter construct was based on the already published predicted structure by Alphafold^38^ and visualized using UCSF Chimera,^39^ illustrated in Figure 2B. Our rationale for delineating the N-terminal domain prioritized retaining its native fold and consequent function. As such, recombinant M1-A172 SELU spanned the first 172 residues of the full-length protein, terminating shortly in the intrinsically disordered linker region at alanine. A hydrophobic side chain at the C-terminus would be less likely to fold into the primary structure,^40^ while its small size would not sterically hinder the local secondary structure. The 10-carbon geranyl group (Figure 2A) has been shown to have an ideal chain length to stabilize the U:G pair by fitting into a minor-like groove of the tRNA–mRNA duplex.^41^ This implicitly questions whether the same geranyl group is also recognized by M1-A172 SELU. Therefore, in addition to simamycin, we also used ungeranylated 2-thiouridine (Figure 1.2) and geranyl pyrophosphate (GePP) as the control to test the binding affinity with M1-A172 SELU since they are the natural substrates for the two-step SelU-catalyzed reaction.

The intrinsic fluorescence of W83, highlighted in navy blue near the interdomain cleft (Figure 2B), was exploited as a hypothetical catalytic residue to determine the binding and thermodynamic parameters. The only other tryptophan, W110 (turquoise), is less solvent-exposed and orientated away from the interdomain cleft, raising doubts on its quenching propensity and proximity to the suspected active site, respectively. Since tryptophan is a polar residue, we noted nearby polar residues presenting additional sites for potential hydrogen bonding and dipole interactions. Residues within 6 Å of W83 are highlighted as N44 (red), R79 (green), D81 (yellow), R84 (orange), Q89 (purple), N90 (pink), and W110 (turquoise). Namely, the side chains of N44, R79, and D81 appear likely to form a salt bridge involved in substrate binding. Interestingly, the purported binding residue C97 (black) is just beyond this vicinity, though conformational rearrangements may bring it closer for its potential role in the selenation reaction.

Titration of M1-A172 SELU with simamycin, plotted in Figure 3A, led to quenching of fluorescence emission, connoting W83 as an active site residue or indicating a conformational change. From the saturation-binding isotherm data shown in Figure 3B, equilibrium dissociation constants (*K*_d_) were calculated at 5 °C (12.60 μM), 12 °C (18.22 μM), 20 °C (29.89 μM), and 40 °C (67.99 μM). Abrogating potential contributions from collisional quenching, the positive correlation between these variables indicated a static quenching mechanism, thereby validating the formation of a ground-state complex. A corresponding van’t Hoff Plot was constructed accordingly, as shown in Figure 3C, in order to obtain the change in enthalpy and, in turn, calculate the changes in entropy and free energy associated with this complex formation. Alternately, in the case of limited material, one can use the van’t Hoff equation to derive this same value based on only two temperature series. In this case, the binding constants were substituted from the lowest and highest series (*T*_1_ = 278 K; *T*_2_ = 313 K; *K*_1_ = 7.94 × 10^4^ M^–1^; *K*_2_ = 1.47 × 10^4^ M^–1^) in order to deduce an identical enthalpy value (−34.885 kJ mol^–1^) as that extracted from the plot. Tabulated in Figure 3E, the negative values across the board indicate a spontaneous (−25.573 kJ mol^–1^) and energetically favorable (−34.996 kJ mol^–1^ K^–1^) binding event, resulting in a more ordered state (−32.244 J mol^–1^ K^–1^). The latter is reminiscent of the two separate molecules adopting their bound orientation, manifested by a loss of degrees of freedom. The signs and magnitudes of these thermodynamic parameters were used to intuit the predominant noncovalent interaction types characteristic of this complex. Consistent with the nucleoside template of simamycin, shown in Figure 3D, and the hydrophilic residues around W83, the binding event was most likely attributed to van der Waals interactions and hydrogen bonding.

**Figure 3:**
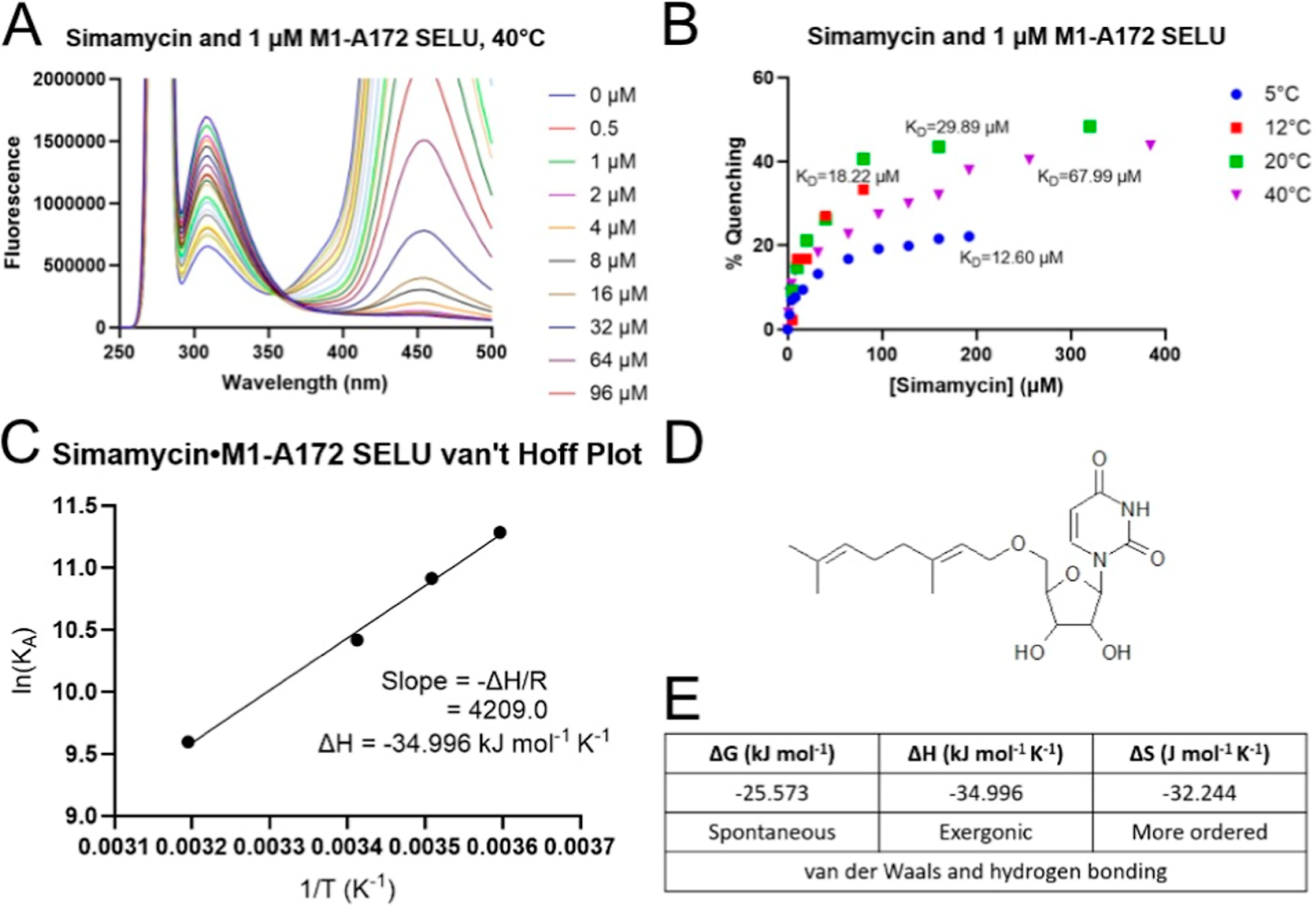
Simamycin and M1-A172 SELU interactions. (A) Fluorescence change when M1-A172 SELU was titrated with 0.5–96 μM simamycin at 40 °C and (B) equilibrium dissociation constants at 5, 12, 20, and 40 °C that were extracted from the fitted saturation-binding isotherms. (C) Δ*H* value for this complex formation was derived from the van’t Hoff plot, and corresponding thermodynamic values (*E*) were calculated in order to infer the predominant noncovalent interaction types. (D) The molecular structure of simamycin.

A Stern–Volmer plot and a double-log Stern–Volmer plot were further used to determine the bimolecular quenching rate constant (*k*_q_) and number of ligand-binding sites, respectively. Plotted in Figure 4A, the values of *k*_q_ across the temperature series fall below 0.5 M^–1^s^–1^, well short of the maximum diffusional quenching rate of 2 × 10^10^ M^–1^s^–1^.^42^ Though diffusion-controlled reactions are indicated by a smaller *k*_q_, such a value can also describe broader conformational changes. Here, this slow rate can be accounted for by a slow mechanism of catalysis typical of interdomain movements. Such broad conformational rearrangements would be permissible due to the 18-residue interdomain linker and even required for enzymatic catalysis if S-geranyl-tRNA and geranyl pyrophosphate indeed bind to separate domains. Furthermore, given the predominant interaction types as van der Waals forces and hydrogen bonding in addition to the O-geranyl group on simamycin, two binding sites should be detected if both native ligands—2-thiouridine and geranyl pyrophosphate—bind to the N-terminal domain of SelU. As shown in Figure 4B, a single binding site (*n*) was determined for M1-A172 SELU from the slope of the double-log Stern–Volmer plot, with that value skewing lower at the two higher temperatures.

**Figure 4:**
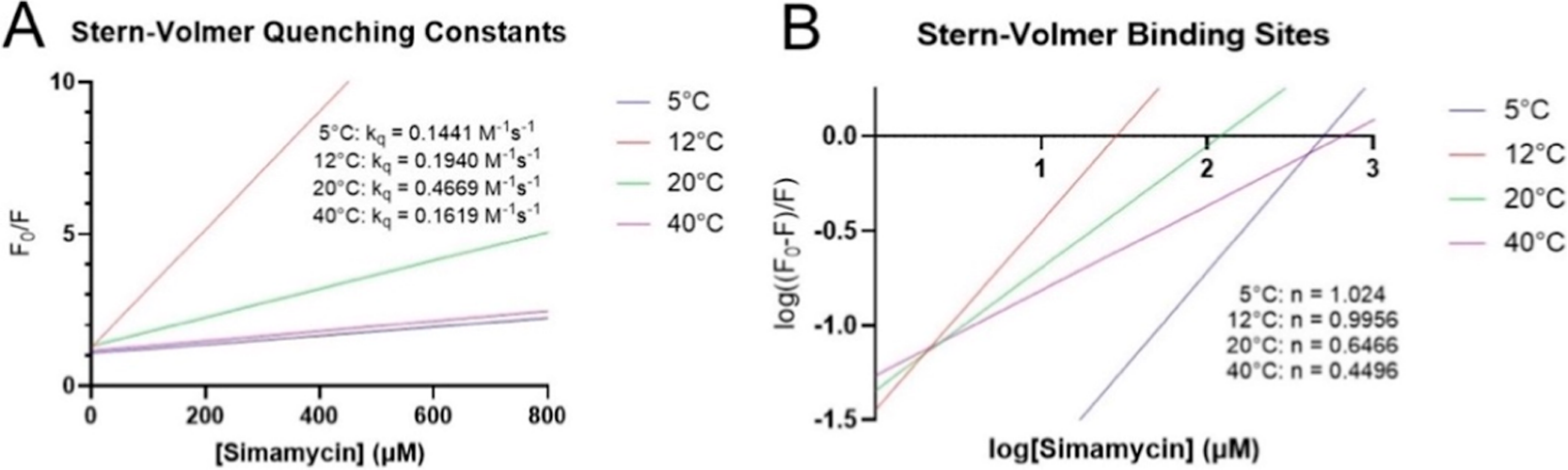
Simamycin and M1-A172 SELU Stern–Volmer plots. Linearized Stern–Volmer plots were constructed in order to determine the bimolecular rate constant at each temperature for this binding event (A) and the number of analyte binding sites (B).

This led us to test whether the isoprenyl group had any affinity to the M1-A172 SELU by separately titrating with thiouridine and geranyl pyrophosphate, illustrated in Figure 5. Despite the former containing several hydrogen bond donors and acceptors as well as being rich in dipole moments conducive to van der Waals interactions, 2-thiouridine failed to attenuate the fluorescence of M1-A172 SELU across a similar titration range used with simamycin. The same lack of affinity toward M1-A172 SELU was found with geranyl pyrophosphate, thereby excluding the binding site of this native substrate to the C-terminal domain.

**Figure 5:**
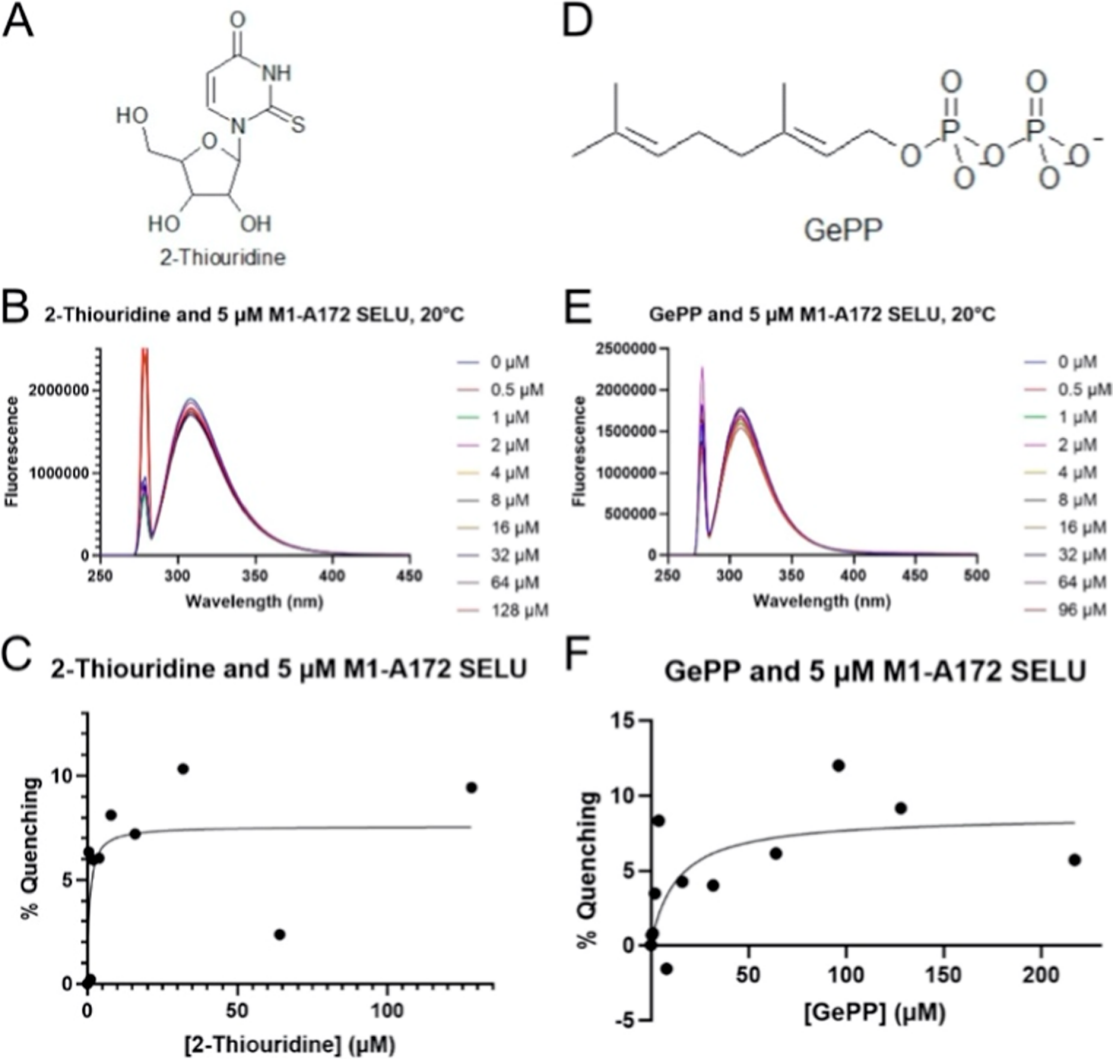
2-Thiouridine and geranyl pyrophosphate lack affinity to M1-A172 SELU. Titration of M1-A172 SELU with 2-thiouridine (A–C) and geranyl pyrophosphate (C–E) demonstrated a lack of affinity of either ligand for the analyte. Fluorescence remained largely unaltered upon addition of either ligand (B,E), reflected by randomness within their respective saturation-binding isotherms (C,F).

The failure of both 2-thiouridine and geranyl pyrophosphate to bind M1-A172 SELU underscores the complexity of this two-step catalysis, involving a mechanistic role of the geranyl group in the tRNA binding event. During such cooperativity, a divalent magnesium cation likely chelates a tRNA-2-thiouridine to the active site of the N-terminal domain: Szczupak et al. found 10 mM magnesium sulfate to support optimal enzymatic activity.^27^ Binding of geranyl pyrophosphate to the C-terminal domain would then elicit an interdomain transfer of the geranyl group afforded by an 18-residue flexible linker, leaving the pyrophosphate byproduct free to regenerate ATP for the selenophosphate synthetase-mediated generation of selenophosphate. Hydrophobic interactions arising from the geranylation of the native 2-thiouridine substrate likely cause a local conformational rearrangement, thereby exposing charged and polar residues of the active site, highlighted in Figure 2B, that bind the nucleoside via the calculated hydrogen bonding and dipole interactions. The 2-thiogeranyl-tRNA is now primed to insert its terpene unit into the minor groove of the duplex and undergo subsequent selenation. We believe that the geranyl substituent on simamycin supersedes this mechanism to directly hijack the tRNA binding site.

To identify the origin of these hydrophobic interactions, we characterized simamycin using a series of homonuclear NMR experiments, shown in Figure 6A, and probed its interacting moieties with M1-A172 SELU via saturation difference transfer (STD) NMR,^43^ illustrated in Figure 6B,C. STD NMR identifies protons of a small-molecule ligand that are in the immediate proximity of its target protein binding site. These protons are observed as strong peaks in the STD spectrum (Figure 6C) versus those in the reference spectrum (Figure 6B). Normalized intensities of the STD peaks, displayed as a group epitope map (GEM) in Figure 6D, reflect the distances of the simamycin protons to the M1-A172 SELU (Figures 6B,C). Owing to the difference in buffer composition between the simamycin sample and that used in the STD experiments, deviations among the small molecule’s chemical shifts were observed (Figure 6A–C). Nonetheless, the greatest contributions to binding and complex formation are from the H2 proton of the geranyl group and H4′ and H5′ protons from the ribose sugar, validating the importance of these two moieties in our simamycin pharmacophore and underscoring their roles in our proposed mechanism of catalysis. Moreover, interactions from the latter protons validate the calculated dipole contributions to binding, as both H4′ and H5′ have adjacent nucleophilic oxygens.

**Figure 6:**
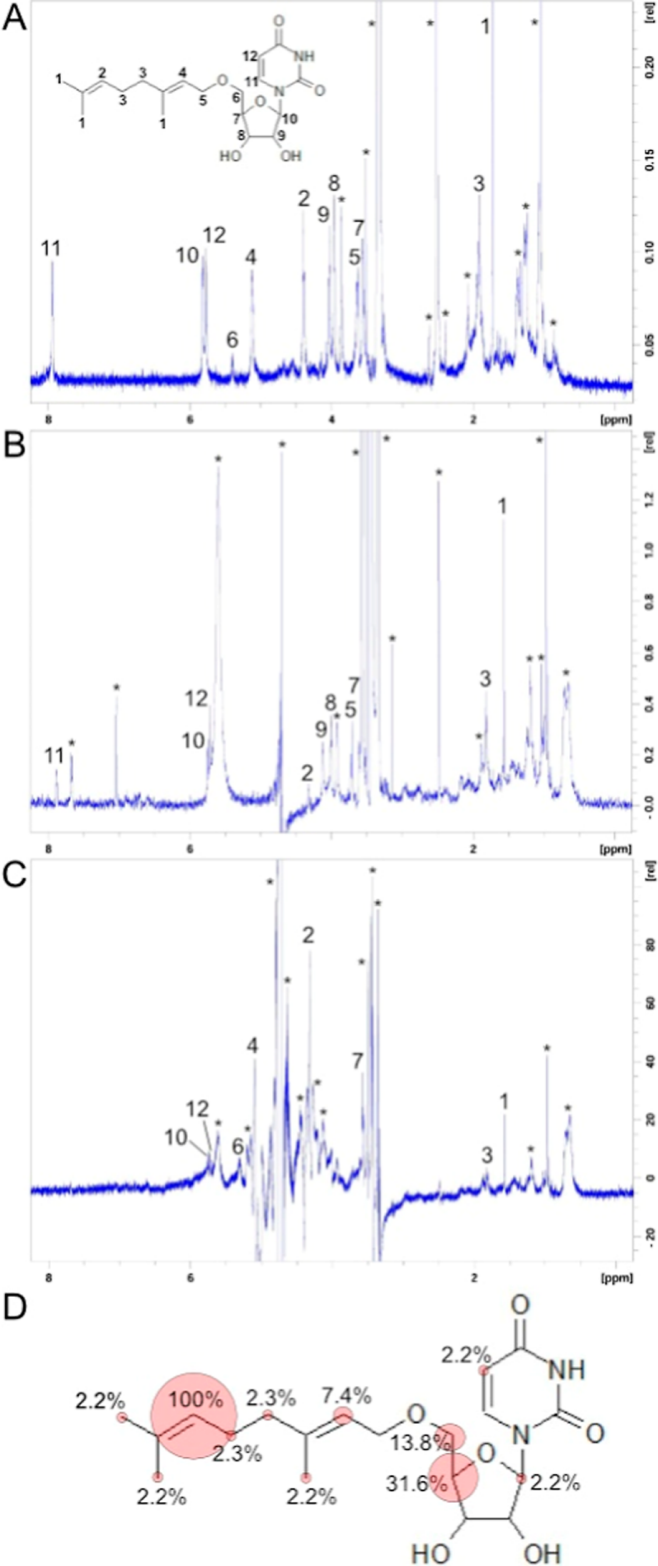
STD NMR and GEM of simamycin and M1-A172 SELU. Proton assignments of simamycin were made using a ^1^H spectrum and homonuclear TOCSY and NOESY spectra (A). Peaks originating from the buffer or impurities are annotated with asterisks (A–C). A separate reference spectrum of simamycin was collected (B) in STD NMR buffer, resulting in salinity-induced deviations among chemical shifts from their positions of original assignment. The peaks of the STD spectrum (C) were integrated in order to quantify the binding contribution from each hydrophobic proton, which was normalized and subsequently illustrated in a group epitope map (GEP) (D).

## Conclusion

Nature has selected 20 amino acids and 4 nucleic acids as building blocks for the macromolecules that carry out all cellular processes. Beyond these canonical molecules, variability among the chalcogens, namely, sulfur and selenium, can imbue the modified macromolecules with unique properties. For instance, the lower electronegativities of cysteine and selenocysteine increase their reactivities. Likewise, selenium substitution at the tRNA 34 position imparts higher hybridization and enhances certain codon-anticodon interactions. In fact, as manufacturers of proteins, tRNAs are the most commonly modified nucleic acids. Their substitution at the wobble position is catalyzed by an enzyme-dubbed tRNA-2-selenouridine synthase in an ATP-dependent process. The structural and mechanistic underpinnings of this bacterial enzyme’s function may provide an avenue for antibiotic development.

Our biophysical investigation into the small geranylated natural product simamycin and the N-terminal SelU construct M1-A172 SELU sheds light on the general composition of the tRNA-SelU binding interface and mechanism of catalysis. Simamycin shows great affinity to M1-A172 SELU, involving both hydrophobic interactions and hydrogen bonding provided by the geranyl group and uridine, respectively. However, neither the thionucleoside nor the geranyl group alone was adequate in binding M1-A172 SELU, suggesting a cooperative mechanism of catalysis involving interplay among the domains as well as a potential local conformational rearrangement around the tRNA-binding site. Reinforcing the notion of distinct binding sites on each domain is the single binding site on the M1-A172 SELU determined by the double-log Stern–Volmer plot, precluding the geranyl pyrophosphate binding site to the C-terminal domain. The affiliation of a pregeranylated 2-thiouridine-tRNA with the N-terminal active site is likely due to a chelating effect with a divalent magnesium cation. Given the role of the geranyl group in initiating the noncovalent interactions of tRNA and the active site, the 18-residue interdomain linker might bring the geranyl group into proximity with the tRNA for a transfer reaction.

Biologically, the now S-geranylated tRNA can insert its terpene group into the minor groove of a U/G containing short duplex in codon–anticodon recognition or remain bound to SelU for subsequent selenation. In the latter case, ensuing hydrophobic interactions between the S-geranylated tRNA and hydrophobic residues of the active site would elicit a local conformational rearrangement. Consequent exposure of polar and hydrophilic side chains would cater to van der Waals interactions and hydrogen bonding with the ribonucleoside, leaving its S-geranyl group solvent-exposed for replacement with selenium.

We believe that simamycin circumvents the native cooperativity among the domains owing to its prepackaged geranyl group, which carries the hydrophobicity necessary for inducing the proposed active site rearrangement. Consistent with its formation of a stable ground state complex with M1-A172 SELU is the positive relationship between the equilibrium dissociation constant and temperature, repudiating any supposed collisional quenching. As such, bimolecular quenching rate constants well below the threshold of a static quenching mechanism authenticate our assertion of a conformationally dynamic binding mode. For instance, the strong affinity of simamycin to M1-A172 SELU may be explained by the O-geranyl group removing hydrophobic side chains from the tRNA active site, corroborated by our STD NMR analysis, thus allowing for the calculated dipole interactions and hydrogen bonding between the ribonucleoside and its target.

Of course, our assertions about an allosteric mode of catalysis can only be experimentally validated through spin-relaxation and chemical exchange experiments, which would provide insight into the relative movement of each residue on the picosecond to millisecond time scales. Those exhibiting flexibility in the bound state can be deemed to participate in catalysis directly or allosterically. A requisite to surveying its molecular dynamics is elucidating the solution structure of M1-A172 SELU. Forbearing knowledge of the enzyme’s structure, ligand-based means of therapeutic development suffice. Thus, simamycin provides a good starting molecular framework that can be further optimized into potential antibiotics targeting SelU through additional chemical modifications.

## Materials and Methods

### Chemical Synthesis

Simamycin has been chemically synthesized following the well-validated route by Igarashi and co-workers.^37^ All analytical data of the compound that we handled fully matched those reported in the literature.

### Gene Cloning and Plasmid Transfection

A gene-insert encompassing residues M1-A172 of SELU_ECOL5, UniProt entry Q0TKD8, with a His(6×)- and FLAG-tags along with a TEV protease cleavage site appended to the N-terminus, was cloned into a pET16b plasmid vector per GenScript. This plasmid was transfected into chemically competent E. coli BL21­(DE3), and transformed colonies were grown overnight at 37 °C and stored at 4 °C for up to one month.

### M1-A172 SELU Expression in BL21­(DE3)

Several colonies of BL21­(DE3) were inoculated into 5 mL of Luria broth (LB) with 100 μg/mL ampicillin and grown overnight in an incubator–shaker at 37 °C and 225 rpm. The following day, this starter culture was decanted into 1 L of LB with 100 μg/mL ampicillin and grown until an optical density of 0.6–0.9 was attained at 600 nm. The lac repressor was removed with 1 mM allolactose analogue isopropylthio-β-galactoside (IPTG), and overexpression of the recombinant protein commenced for 16–20 h at 25–30 °C.

### M1-A172 SELU Purification

The cells were harvested at 2,500 g for 20 min and resuspended in 20 mL of lysis buffer consisting of 50 mM Tris, pH 8.0, 500 mM NaCl, 20 mM imidazole, 7 mM βME, and 8 M urea. Cell lysis occurred via five 3 min cycles of sonication at a 50% amplitude and one second pulse. The cell lysate was nutated at 4 °C for 30 min in order to allow the chaotropic agent to electrostatically disrupt the inclusion bodies. It was then centrifuged at 17,000 g for 1 h at 4 °C, and the 20 mL supernatant was loaded three times onto 4 mL of nickel resin at 4 °C. The column was washed with 100 mL of 20 mM imidazole, 500 mM NaCl, 50 mM Tris, pH 8.0, and eluted in 20 mL of RNA hydrolysis buffer (25 mM Tris, pH 7.60, 20 mM NaCl, 5 mM BME, and 3.5 M urea—spiked with 300 mM imidazole. To this eluate was added 0.75 mg of RNase A, and RNA hydrolysis ensued for 2 h at 37 °C. The tRNA-free construct was then “salted out” by saturating the solution with ammonium sulfate, and the precipitated protein clusters were pelleted by centrifuging for 30 min at 17,000 g and room temperature. RNase A did not “salt out” and was thus discarded from the supernatant. Resolubilizing the pelleted recombinant protein precipitate required high salt and chaotropic agents, so double the concentration of TEV digestion buffer—25 mM Tris, pH 8.0, 150 mM NaCl, 5 mM βME, and 2 M urea—was added to the pellet in a 10 mL volume. Once the protein was resolubilized, the volume was doubled in order to obtain the stated concentrations, and overnight TEV digestion proceeded in a 50 mL Falcon tube with gentle rocking at 4 °C. The now untagged M1-A172 SELU was separated from His­(6x)-TEV by passing the sample through a subsequent nickel column, with pure M1-A172 SELU collected in the flowthrough. The sample was concentrated to 1 mL using a 15 mL Amicon filter with a 10 kDa MW cutoff and eluted from a Sephacryl S-300 HR size-exclusion column in experimental buffer containing 10 mM sodium phosphate, pH 7.20, and 100 mM sodium chloride. To this pure sample, which could be stored for up to a month at 4 °C, were added 1 mM TCEP and 0.1 mM AEBSF.

### Fluorescence Spectroscopy

A Fluorolog-3 fluorescence spectrophotometer with an excitation wavelength set to 280 nm and a spectral width from 250 to 500 nm recorded fluorescence emission of M1-A172 SELU with varying ligand concentrations in a 500 μL quartz cuvette. Specifically, 1–5 μM M1-A172 SELU were titrated with 0.5 μM to 2000 μM ligand in experimental buffer, and 5 min incubation times allowed equilibrium to be reached prior to each acquisition.

The concentration of the ligand was plotted against percent quenching for each temperature series, 5–40 °C, and the equilibrium dissociation constant, *K*_D_, was determined by fitting the saturation binding curve to Prism’s “one-site, specific binding” equation.

A van’t Hoff plot was subsequently constructed for each temperature series by plotting the natural logarithm of the equilibrium association constant, *K*_A_, against the inverse temperature in units of inverse Kelvin. The enthalpies of formation were obtained from the slopes of these graphs, and Gibbs free energy and the change in entropy associated with these complex formations were calculated from the respective equations, Δ*G* = −RT*ln­(*K*_A_) and Δ*G* = Δ*H* – *T*Δ*S*. The signs and magnitudes of these thermodynamic parameters and their documented correlations were used to infer the types of noncovalent interactions presiding over each receptor–ligand complex.

The bimolecular quenching rate constant was derived from the Stern–Volmer quenching constant, *K*_SV_ = *K*_q_τ_0_, where τ_0_ is 10^–8^ s, and was discerned for each receptor–ligand complex by comparing the quenching rate constant to the maximum diffusion collisional quenching rate constant of 2.0 × 10^10^ M^–1^s^–1^. Moreover, the stability of each complex was indicated by the changing values of *K*_A_ with temperature.

The number of receptor binding sites was determined by fitting the data to the double-log Stern–Volmer equation, log­((*F*_0_ – *F*)/*F*) = log­(*K*_A_) + *n* log­[*Q*], where *F* and *F*_0_ are the fluorescence intensities with and without quencher (*Q*), respectively, and n is the parameter of interest.

### Saturation Transfer Difference Nuclear Magnetic Resonance Spectroscopy

A Bruker AVANCE II 700 MHz spectrometer equipped with a TXI cryoprobe was used to acquire the ^1^H spectrum of simamycin at room temperature. Homonuclear ^1^H–^1^H TOCSY and ^1^H–^1^H NOESY spectra were acquired to aid in the proton assignments. NMR samples contained 1 mM simamycin in 100% deuterated-DMSO. The NMR sample for STD NMR experiments contained 30 μM M1-A172 SELU and 300 μM simamycin in 60% D_2_O, 10 mM potassium phosphate, pH 7.3, and sodium chloride. The experiment was performed at 25 °C.

As per the protocol of Mayer et al.,^43^ the STD NMR data set was acquired using a T_1ro_ filter to suppress signals from a protein target. A train of 40 Gaussian-shaped pulses, each 50 ms long at 40 dB and separated by a 1 ms delay, were used to saturate protein proton resonances. The off-resonance irradiation was applied at 28,000 Hz, where no protein proton signals were present. The on-resonance spectrum was applied by irradiating the protein at a carrier frequency of 0.06 ppm, corresponding to 41.91 Hz. Subtraction of on-and-off resonance spectra following each scan via phase cycling yielded the difference spectrum. A group epitope map (GEM) was constructed by integrating the peaks of the difference spectrum and normalizing the greatest value to 100%.

## Supplementary Material



## Notes

Primary structure of M1-A172 SELU, W83 as the internal probe, and synopsis of simamycin proton chemical shift assignments (PDF)
