# Supplementary material for: A Geranylated Natural Product Simamycin Disrupts the Allosteric Catalysis of tRNA-2-selenouridine Synthase SelU
Source: Biochemistry. 2025 May 26;64(12):2640–8. doi: 10.1021/acs.biochem.5c00053 (PMC12177912; doi:10.1021/acs.biochem.5c00053)
Supplement: Supplementary file 1 [file bi5c00053_si_001.pdf]

Supplementary Information

A Geranylated Natural Product Simamycin Disrupts the Allosteric Catalysis of tRNA-2-selenouridine Synthase SelU

Stephen J. Dansereau [1,2], Alexander Shekhtman [1], Francesco Epifano [3], Salvatore Genovese [3], Serena Fiorito [3], Thomas J. Begley [1,4], Jia Sheng [1,2,*]

[1] Department of Chemistry and University at Albany, State University of New York, 1400 Washington Ave. Albany, NY, 12222, USA.

[2] The RNA Institute, University at Albany, State University of New York, 1400 Washington Ave. Albany, NY, 12222, USA.

[3] Department of Pharmacy, University "Gabriele D'Annunzio" of Chieti-Pescara, Chieti Scalo, 66100, Italy

[4] Department of Biological Science, University at Albany, State University of New York, 1400 Washington Ave. Albany, NY, 12222, USA.

* Corresponding author Email: jsheng@albany.edu

Primary Structure of M1-A172 SELU

MHHHHHHDYKDDDDKENLYFQG

MQERHTEQDYRALLIADTPIIDVRAPIEFEQGAMPAAINLPLMNNDERAAVGICYKQQGS

DAALALGHKLVAGEIRQQRMDAWRAACLQNPHGILCCARGGQRSHIVQRWLHDAGIDYPL

VEGGYKALRQTAIQATIELSQKPIVLIGGCTGCGKTLLVQQQPNGVDLEGLA

W83 as the Internal Probe

The amino acid sequence of M1-A172 SELU contains two tryptophan residues, W83 and W110, highlighted in **Fig 2B**. W83 is believed to be the fluorophore quenched because it is more solvent exposed and resides on the interior of the interdomain cleft, making it accessible to interdomain cooperativity. Though fluorescence correlation spectroscopy and selective isotopic labeling could verify that one or both tryptophan residues are involved in binding, these methods do not specify the residue identity. Doing so would require mutagenesis assays or NMR resonance assignments.

Synopsis of Simamycin Proton Chemical Shift Assignments

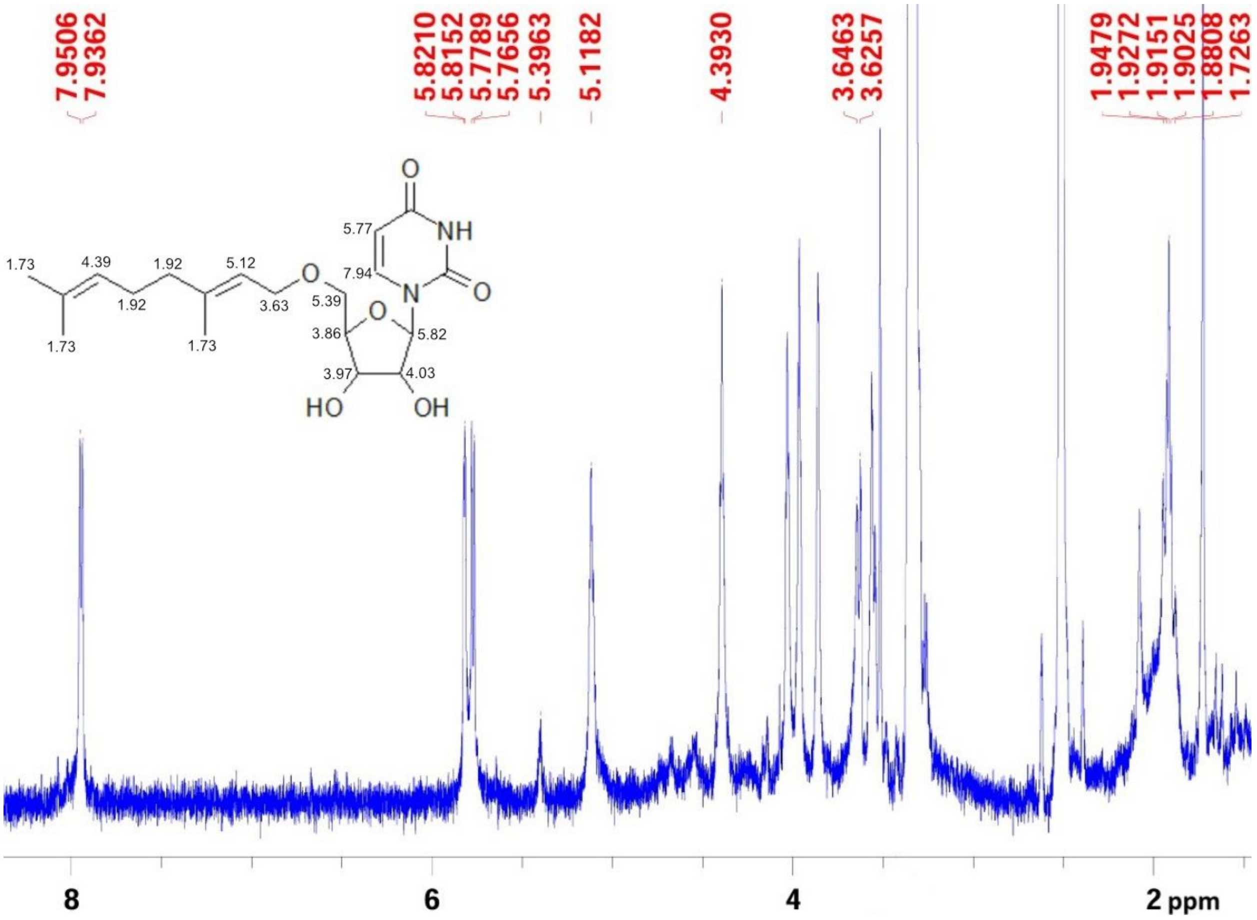

Figure S1: Compound 9 ^{1}H NMR Assignments. Proton assignments were made based on 1D and homonuclear correlation spectra.

Compound 9 contains five total spin systems, defined as a group of protons connected by J-couplings delineated by electronegative atoms or quaternary carbons. The latter theoretically separates the geranyl group into three spin systems, though in practice through-bond correlations were observed throughout the geranyl group until the electronegative oxygen.

The two alkene protons of this geranyl group were shifted slightly upfield of the canonical 5-6 ppm range, at 5.12 and 4.39 ppm, respectively. Both chemical shifts appeared as triplets, in keeping with their J-couplings to two adjacent protons. The alkene group closer to

the electronegative oxygen was assigned to 5.12 ppm while that closer to the methyl group was assigned to 4.39 ppm.

The overlapping quartet and triplet at 1.92 ppm was also correlated through-bond with the alkene protons, and thus assigned to the methylene groups between these protons. The three methyl groups are represented by the singlet at 1.73 ppm, given their absent J-coupling, and shifted downfield from the usual methyl resonance owing to the inductive effects of the adjacent olefins. Still, these methyl groups are through-bond correlated with the protons of the geranyl group.

The doublet at 3.63 ppm was assigned to the methylene group between the alkene and oxygen on the geranyl group. Deshielding of these protons by both the electronegative oxygen and the inductive effect of the alkene resulted in a downfield shift from the typical range of around 1 ppm for methylene protons.

The ribose sugar consisted of a single spin system. Doublets at 5.39 and 5.82 were assigned to the 5’ and 1’ protons, respectively, the latter manifested as deshielding from both oxygen and nitrogen. As with the geranyl group assignments, through-bond correlations within the ribose spin system were observed using the 2D homonuclear TOCSY spectrum. Namely, the quartet at 3.86 ppm, some of the splitting of which is buried in the noise, was assigned to the 4’ proton while the triplets at 4.03 and 3.97 ppm were assigned to the 2’ and 3’ protons.

Through-bond correlations among the doublets at 5.77 and 7.94 ppm revealed the two protons of the nucleobase spin system, the more downfield shift assigned to the 4-position adjacent to the electronegative nitrogen.
